# Topically Applied Magnetized Saline Water Improves Skin Biophysical Parameters Through Autophagy Activation: A Pilot Study

**DOI:** 10.7759/cureus.49180

**Published:** 2023-11-21

**Authors:** Piercarlo Minoretti, Andrés Santiago Sáez, Miryam Liaño Riera, Manuel Gómez Serrano, Ángel García Martín

**Affiliations:** 1 General Direction, Studio Minoretti, Oggiono, ITA; 2 Legal Medicine, Hospital Clinico San Carlos, Madrid, ESP; 3 Legal Medicine, Psychiatry, and Pathology, Complutense University of Madrid, Madrid, ESP

**Keywords:** mtor, beclin-1, cutaneous biophysical parameters, autophagy, magnetized saline water

## Abstract

Background

Water exposed to a magnetic field exhibits several changes in its properties, such as increased electrical conductivity, reduced density, and low surface tension. Additionally, it has reduced dissolved oxygen levels and becomes more alkaline. Previous experimental studies have demonstrated that exposure to saline alkaline water leads to a dose-dependent increase in the expression of autophagy-related genes. Here, we hypothesize that the topical application of magnetized alkaline water to the skin can activate autophagy and improve cutaneous biophysical parameters, making it a promising strategy for enhancing skin aesthetics.

Methods

Two distinct substudies were undertaken. Firstly, a 12-week, uncontrolled, open-label investigation was conducted with 20 females who desired to enhance the appearance of their facial and neck skin. Secondly, a molecular study was carried out on a subset of 10 females to investigate the serum’s impact on two autophagy markers (Beclin-1 and mammalian/mechanistic target of rapamycin {mTOR}) in skin biopsies taken from the posterior neck area below the hair attachment line.

Results

After a period of 12 weeks, the application of the serum resulted in significant improvements in skin hydration within the stratum corneum (56 ± 14 arbitrary units {a.u.}) compared to the baseline measurement (47 ± 12 a.u.; p < 0.001). Moreover, the transepidermal water loss (TEWL) decreased from 14 ± 2 g/m^2^/hour to 11 ± 3 g/m^2^/hour (p < 0.001). The results also revealed a notable reduction in sebum content from 38 ± 7 µg/cm^2^ to 30 ± 4 µg/cm^2^ after the 12-week period of serum application (<0.001). Additionally, the melanin index (p < 0.01) and erythema index (p < 0.001) were both significantly lower at 12 weeks compared to baseline. The molecular study showed a 38% increase in Beclin-1 levels after 12 weeks of serum application on the posterior neck area, as measured from skin biopsies. In contrast, mTOR levels decreased by 24% from baseline to 12 weeks.

Conclusion

The application of magnetized saline water topically, within a serum formulation, shows potential in improving skin biophysical parameters for females seeking to enhance the appearance of their facial and neck skin. These beneficial effects are achieved through the activation of cutaneous autophagy, as evidenced by an increase in Beclin-1 expression and a decrease in mTOR content in the skin.

## Introduction

The human skin is constantly exposed to a multitude of stressors that can negatively impact its health and appearance. Factors such as climate change [1], environmental pollutants [2], sunlight [3], and imbalances in the skin’s microbiota [4] can accelerate the development of undesirable cutaneous alterations. These modifications often manifest as dryness, changes in texture, an oily complexion, hyperpigmentation, redness, telangiectasia, and keratosis [5-7]. In recent years, there have been extensive efforts to devise innovative skin beautification interventions based on the underlying pathophysiological mechanisms [8].

Growing evidence suggests that autophagy, a cellular process that degrades excess or damaged cell components and recycles cytoplasmic contents [9], plays a significant role in skin homeostasis [10-12]. Significantly, autophagy is involved in processes that can impact the aesthetic appearance of the skin, such as melanogenesis [13], sebaceous gland function [14], and blood vessel formation [15]. In addition, an impaired cutaneous autophagic flux has been associated with cutaneous aging [16]. Taken as a whole, these observations suggest that stimulating skin autophagy could represent an innovative strategy for promoting aesthetic improvement. Moreover, it is widely recognized that maintaining adequate water content in the skin is vital for its proper functioning [17]. Water not only is essential for life and virtually all bodily functions but also plays a crucial role in ensuring the effectiveness of the skin’s barrier and overall envelope function [18]. Importantly, a deficiency in water content has been associated with various cutaneous dysfunctions, such as impaired superficial and deep dehydration, as well as changes in biomechanical properties such as maximum extensibility and the skin’s ability to return to its original state [19]. These alterations can lead to unfavorable changes in the cutaneous physiological properties.

Exposing water to a magnetic field has been found to have several effects on its properties, including alkalinization, increased electrical conductivity, reduced density, low dissolved oxygen, and reduced surface tension [20]. While studies on its potential medical applications are still limited [21,22], a substantial body of evidence has been published on the effects of magnetized saline water, which is typically alkaline [20], on both plants [23,24] and animals [20]. For instance, when aquatic animals are exposed to saline-alkaline water, a dose-dependent increase in the expression of autophagy-related genes has been observed [25]. Conversely, the expression of the mammalian/mechanistic target of rapamycin (mTOR), a key component in regulating cell growth, proliferation, and survival [26], remains persistently subdued under these conditions [5]. Notably, the inhibition of mTOR has recently gained attention as a promising approach to managing both aging-related conditions [27] and skin inflammation [28].

This study explores, for the first time, the potential of magnetized saline water as an aesthetic enhancement ingredient in a topical serum formulation. Based on the known biological effects of magnetized saline water in animals [20,25], we also investigated whether this approach could stimulate skin autophagy, as evidenced by an increased expression of the biomarker Beclin-1 [29], while simultaneously diminishing mTOR expression. To substantiate these hypotheses, we conducted two sets of investigations. First, we performed a 12-week, uncontrolled, open-label study to assess whether the topical application of a serum containing magnetized saline water can enhance the biophysical parameters of facial skin, specifically in the cheek area, in a sample of healthy adult Caucasian females. Following this, we conducted a molecular study aimed at scrutinizing the protein levels of mTOR and the autophagy marker, Beclin-1, in skin biopsies acquired from the serum-treated regions located in the posterior neck.

## Materials and methods

Participants

The study involved 20 Caucasian females who personally recognized the need for the beautification of their facial and neck skin and volunteered to be part of the research. Females who were pregnant, breastfeeding, under 18 years old, or over 65 were ineligible. Additionally, females with immunosuppression, a history of procoagulative or thrombophilic conditions, bleeding or clotting disorders, or a platelet count of less than 100,000/mL were not included. The exclusion criteria also extended to females on anticoagulant therapy or systemic corticosteroids and patients with chronic renal failure, hepatic insufficiency or hepatitis, cardiovascular disorders, diabetes mellitus, thyroid disorders, or cancer. Moreover, females with hemoglobin levels below 12 mg/dL and a history of keloid formation or who had received aesthetic treatments (e.g., chemical peels, dermabrasion, soft tissue filler, botulinum toxin injection, mesotherapy, or resurfacing surgeries) in the past six months were excluded. Individuals concurrently participating in other research projects or clinical trials were also not included. All procedures were performed in outpatient facilities belonging to Studio Minoretti. The study was conducted in accordance with the principles outlined in the Declaration of Helsinki and received approval from the local ethics committee (reference number: SM/AQS/23). Written informed consent was obtained from all subjects prior to their participation.

Procedures

The study involved the use of a facial serum that contains a patented form of magnetized saline water (93% total content) as its sole active ingredient (Aquavis, Brescia, Italy). The participants were instructed to apply the serum each morning for 12 weeks, using a recommended concentration of 2 mg/cm^2^. To ensure comprehensive application, the serum was evenly distributed across the entirety of the face, as well as the posterior and anterior regions of the neck. During the study period, participants were not allowed to use any other topical or systemic treatments.

Clinical study: Skin biophysical parameters

The primary objective of the pilot, open-label, clinical study was to evaluate the impact of the facial serum on six distinct skin biophysical parameters measured on the cheek area. We conducted pre- and post-application assessments over a 12-week period using an array of probes attached to an MPA10 MultiProbe Adapter System (Courage & Khazaka Electronic GmbH, Cologne, Germany) [30], which was connected to a computer. Skin hydration in the stratum corneum was gauged using the Corneometer CM825 (Courage & Khazaka Electronic GmbH), which relies on capacitance measurements. The results were expressed in arbitrary units (a.u.), and each measurement was an average of at least five readings. The epidermal barrier function was evaluated by measuring the transepidermal water loss (TEWL) using a Tewameter TM300 (Courage & Khazaka Electronic GmbH). The average was taken from at least three readings. Sebum content was measured with Sebumeter SM 815 (Courage & Khazaka Electronic GmbH). Lastly, we used the Mexameter MX18 (Courage & Khazaka Electronic GmbH) to evaluate the skin’s melanin and erythema indexes, again taking the average from at least three readings. All measurements were conducted in the same anatomical areas and under consistent environmental conditions to ensure the accuracy and reliability of the results.

Clinical study: Secondary endpoints

The secondary endpoints of the open-label study were to assess tolerance and overall satisfaction related to the utilization of the facial serum. To gauge tolerance, inquiries were made to patients regarding any localized symptoms such as burning, itching, or stinging sensations, alongside any systemic adverse reactions. Furthermore, satisfaction with the cosmetic results of the serum was assessed using a four-point scale, which included the following categories: excellent, good, average, and poor.

Molecular study

Among the 20 study participants, a subgroup of 10 females volunteered to take part in a molecular investigation. We obtained 4 mm^2^ punch biopsies from these participants both before and after treatment. The procedure was conducted on the serum-treated posterior neck area, beneath the hair attachment line. This location was strategically selected to minimize any potential aesthetic concerns, guaranteeing that the biopsied area would remain discreet. The tissue obtained was thoroughly homogenized, and in skin homogenates, we used two commercial enzyme-linked immunosorbent assay (ELISA) kits (MyBioSource, Inc., San Diego, CA) to quantify concentrations of Beclin-1 and mTOR. To determine individual concentrations, we established standard curves for each biomarker and converted the mean fluorescence intensity from each well into a concentration using the linear portion of the standard curve. The results were expressed in arbitrary units (a.u.), with baseline values set at 100 prior to the application of the product. Each measurement was performed twice and averaged. The intra- and inter-assay coefficients of variation were below 7% and 9%, respectively.

Data analysis

We used descriptive statistics to express the results. To compare pre- and posttreatment data, paired Student’s t-tests were conducted. The relationships between the study variables were examined using Spearman’s correlation coefficient. Analyses were conducted using the Statistical Package for Social Sciences (SPSS) version 20.0 (IBM SPSS Statistics, Armonk, NY). All hypothesis testing was two-sided, and statistical significance was set at p < 0.05.

## Results

Clinical study: Skin biophysical parameters

The study sample comprised 20 females with a mean age of 37.6 ± 8.1 years (range: 22-58 years). All participants successfully completed the study. The biophysical parameters measured in the cheek skin before and after the 12-week application period of the facial serum are summarized in Table 1.

**Table 1:** Changes in skin biophysical parameters from baseline to 12 weeks in the study participants (n = 20) Data are expressed as means ± standard deviations SC, stratum corneum; TEWL, transepidermal water loss; a.u., arbitrary units

Variable	Baseline	12 weeks	P value
Skin hydration in the SC, a.u.	47 ± 12	56 ± 14	<0.001
TEWL, g/m^2^/hour	14 ± 2	11 ± 3	<0.001
Sebum content, µg/cm^2^	38 ± 7	30 ± 4	<0.001
Melanin index, a.u.	142 ± 39	134 ± 26	<0.01
Erythema index, a.u.	251 ± 45	210 ± 46	<0.001

**Figure 1:**
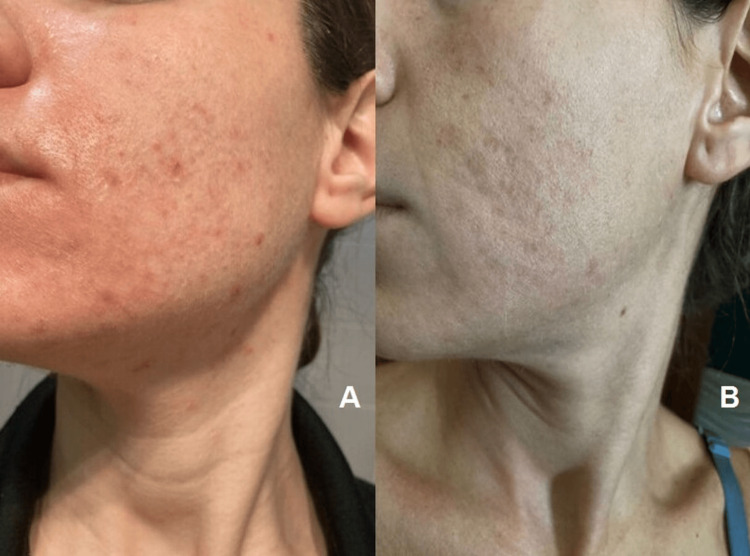
Illustrative images of the visible reduction in skin oiliness and acne lesions from baseline (panel A) to 12 weeks (panel B) induced by a topical lotion containing magnetized saline water

After 12 weeks, skin hydration within the stratum corneum significantly (p < 0.001) increased (56 ± 14 a.u.) from baseline (47 ± 12 a.u.). Concurrently, TEWL decreased from 14 ± 2 g/m^2^/hour to 11 ± 3 g/m^2^/hour, respectively (p < 0.001). These findings suggest an overall improvement in the skin barrier function. Notably, we also found a marked reduction in sebum content from 38 ± 7 µg/cm^2^ to 30 ± 4 µg/cm^2^ (p < 0.001; Figure 1) after 12 weeks of serum application.

**Figure 2:**
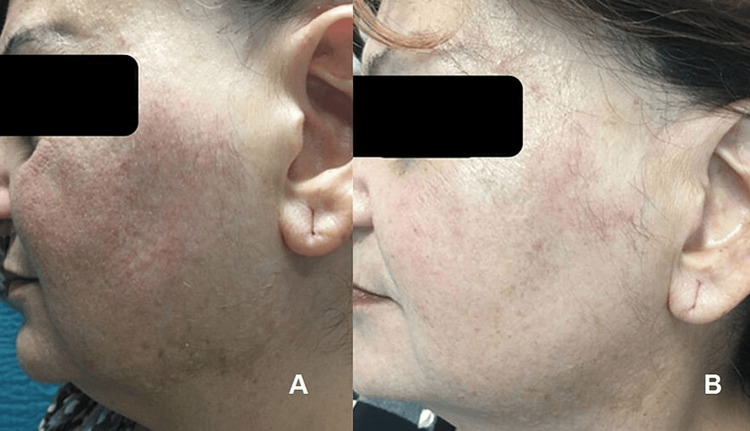
Illustrative images of the visible reduction in cheek erythema and melasma lesions in the skin over the inferior border of the mandible from baseline (panel A) to 12 weeks (panel B) induced by a topical lotion containing magnetized saline water

Additionally, the melanin index was notably lower at 12 weeks compared to baseline (142 ± 39 a.u. compared to 134 ± 26 a.u., respectively; p < 0.001), as was the erythema index (251 ± 45 a.u. compared to 210 ± 46 a.u., respectively; p < 0.001; Figure 2).

Clinical study: Secondary endpoints

The facial serum containing magnetized saline water was well-tolerated by all participants, and none of them reported any adverse local or systemic effects. There were no reports of itching, burning, or stinging sensations. In terms of satisfaction with the cosmetic results, 10 females (50%) rated the product as excellent, six (30%) as good, three (15%) as average, and one (5%) as poor.

Molecular study

In the molecular study involving 10 females, Beclin-1 levels increased by 38% at 12 weeks (138 a.u.) in the serum-treated area on the posterior neck, relative to the baseline (set at 100 a.u.) as quantified from skin biopsies. Concurrently, mTOR levels decreased by 24% from baseline (set at 100 a.u.) to 12 weeks (76 a.u.). Significant correlations were observed between the increase in Beclin-1 levels in skin biopsies and various biophysical parameters, including skin hydration (Spearman’s rho = 0.47; p < 0.05), TEWL (Spearman’s rho = -0.51; p < 0.05), melanin index (Spearman’s rho = -0.42; p < 0.05), and erythema index (Spearman’s rho = -0.56; p < 0.05). These associations suggest that an increased expression of Beclin-1 is linked to an increase in skin hydration and a decrease in TEWL, melanin index, and erythema index. Additionally, mTOR levels in skin biopsies were found to decrease in parallel with sebum content (Spearman’s rho = 0.55; p < 0.05), indicating that a reduction in mTOR was related to an improvement in the oily complexion.

## Discussion

Our study provides evidence that the use of a facial serum containing a patented form of magnetized saline water can effectively enhance skin biophysical parameters in females seeking improvement in their facial and neck skin. Not only was the treatment approach found to be safe and well-tolerated, but also it garnered high levels of satisfaction among the study participants. Furthermore, our molecular investigations revealed that the serum’s beautification effects are directly linked to the regulation of two crucial autophagy-related molecules, namely, Beclin-1 and mTOR. Specifically, our findings indicate that the application of the serum leads to an upregulation of Beclin-1 expression, resulting in improved skin hydration and decreased TEWL, melanin index, and erythema index. Conversely, the serum’s ability to reduce skin sebum content can be attributed to the downregulation of mTOR levels.

Beclin-1 plays a crucial role in regulating autophagy activity by inducing the maturation of autophagosomes and phagosomes, along with the regulation of recycling endosomes [31]. Beclin-1 is also involved in maintaining skin hydration and regulating TEWL. Specifically, Noguchi et al. [32] have demonstrated that Beclin-1-deficient mice exhibit significantly higher TEWL levels, indicating the importance of Beclin-1 in the generation of the skin barrier. In Beclin-1 knockout mice, dysfunctional integrin recycling appears to be the primary cause of skin developmental defects in the epidermis. These findings emphasize the crucial role of Beclin-1 in preserving the integrity of the barrier function [32]. Furthermore, inhibiting autophagy by depleting Beclin-1 can effectively hinder the proliferation and differentiation of keratinocytes [32]. Based on these findings, Kim et al. [33] proposed autophagy as a pivotal guardian of the skin barrier.

Our study also revealed a significant correlation between the increase in skin Beclin-1 levels, triggered by a 12-week application of the facial serum, and a decrease in both the melanin and erythema indexes. Prior research has indicated that autophagy may play a role in regulating skin color by controlling melanosome degradation in keratinocytes [13]. This suggests that inducing autophagy could potentially lead to a skin-whitening effect [34]. The overaccumulation of melanin in the skin can cause hyperpigmentation disorders such as melasma [35]. While the majority of hypopigmentation treatments have been designed to target tyrosinase, it is noteworthy that tyrosinase inhibitors have been found to have limited clinical efficacy and can cause potential adverse effects [36]. As a result, there is a need for innovative strategies to achieve anti-melanogenic effects.

Autophagy inducers, anticipated to be safe, such as magnetized saline water, hold promise as new and viable alternatives for reducing melanin pigmentation. Regarding the inverse relationship between levels of Beclin-1 and the erythema index, previous research by Van Hove et al. [37] has demonstrated that autophagy in keratinocytes can reduce skin inflammation. In an animal model deficient in keratinocyte autophagy, the authors observed a significantly increased inflammatory response, which resulted in more erythema and scaling compared to control animals [37]. Another possible explanation for the link between autophagy activation and decreased erythema could be related to the regulation of vascular tone. Accordingly, prolonged adrenergic stimulation can hinder basal autophagy and trigger endoplasmic reticulum stress in the vascular endothelium [38]. This stress response is crucial for maintaining proper vascular tone. Lastly, our study found that the facial serum containing magnetized saline water reduced mTOR levels in skin biopsies, an effect paralleled by a decrease in skin sebum content. This result is consistent with published evidence that supports the idea that the mTOR axis is involved in the pathophysiology of acne [39]. In patients with acne, both cytoplasmic and nuclear mTOR expression are generally markedly higher than in controls [40]. Notably, Agamia et al. [41] reported a remarkable 20.77-fold increase in mTOR gene expression in the lesional skin of acne patients when compared to skin samples from healthy subjects. Moreover, mTOR expression steadily increases during the transition from a healthy state to lesional acne skin [41].

The precise mechanisms underlying the activation of autophagy by magnetized saline water, typically characterized by its alkaline nature, are currently elusive. Previously, Wang et al. [25] provided evidence suggesting that alkaline stress, induced by saline-alkali water, could maintain mTOR expression in an inhibited state, thereby promoting autophagy. Similarly, Suk et al. [42] reported that the induction of autophagy by alkaline stress is facilitated through the inactivation of mTOR. These findings suggest that the molecular effects observed after facial serum application may be attributed to the localized induction of mild alkaline stress in the skin. Another possibility might be that the transmembrane transport of magnetized saline water within the epidermis is mediated by aquaporin 3 (AQP3), a channel primarily responsible for the transport of water, glycerol, and hydrogen peroxide [43]. AQP3 not only plays a significant role in skin hydration, water retention, and barrier repair but also exhibits the ability to bind to Beclin-1 directly, thereby activating skin autophagy [44]. The question of whether the topical application of magnetized saline water enhances AQP3 expression is yet to be definitively answered. Additional research is warranted to elucidate the molecular mechanisms through which autophagy may be induced by magnetized saline water.

Several caveats of our study warrant discussion. The absence of a placebo arm, the exclusive focus on Caucasian females, and the limited number of participants who underwent skin biopsies are the primary limitations of this research. To gain a more comprehensive understanding, future investigations should include male subjects to facilitate a sex-based analysis of the impact of topically applied serum on skin biophysical parameters. Moreover, it is important to acknowledge that, due to financial limitations, only two autophagy markers were considered. On the one hand, Beclin-1 is involved in the early stages of autophagy, aiding in the creation of autophagic vesicles and recruiting proteins from the cytosol [9]. On the other hand, light chain 3B-II (LC3B-II), which was not included in our study, serves as a marker for the final formation of autophagosomes [9]. While the measurement of Beclin-1 in our study provides valuable insights into autophagy, incorporating the evaluation of LC3B-II in future research is essential. Other areas for future research include investigating the potential adverse effects and response to the prolonged use of products containing magnetized saline water for more than 12 weeks. Additionally, studying the response after discontinuing the serum and including patients of diverse ethnicities and those with preexisting skin conditions such as seborrheic dermatitis, atopic dermatitis, and rosacea would also be valuable avenues for further exploration.

## Conclusions

The application of magnetized saline water topically, within a serum formulation, shows the potential in improving skin biophysical parameters for females seeking to enhance the look of their facial and neck skin. These beneficial effects are achieved through the activation of cutaneous autophagy, as evidenced by an increase in Beclin-1 expression and a decrease in mTOR content in the skin.
